# Identification of epileptic brain states by dynamic functional connectivity analysis of simultaneous EEG-fMRI: a dictionary learning approach

**DOI:** 10.1038/s41598-018-36976-y

**Published:** 2019-01-24

**Authors:** Rodolfo Abreu, Alberto Leal, Patrícia Figueiredo

**Affiliations:** 1 ISR-Lisboa/LARSyS and Department of Bioengineering, Instituto Superior Técnico, Universidade de Lisboa, Lisbon, Portugal; 2 Department of Neurophysiology, Centro Hospitalar Psiquiátrico de Lisboa, Lisbon, Portugal

## Abstract

Most fMRI studies of the brain’s intrinsic functional connectivity (FC) have assumed that this is static; however, it is now clear that it changes over time. This is particularly relevant in epilepsy, which is characterized by a continuous interchange between epileptic and normal brain states associated with the occurrence of epileptic activity. Interestingly, recurrent states of dynamic FC (dFC) have been found in fMRI data using unsupervised learning techniques, assuming either their sparse or non-sparse combination. Here, we propose an *l*_1_-norm regularized dictionary learning (*l*_1_-DL) approach for dFC state estimation, which allows an intermediate and flexible degree of sparsity in time, and demonstrate its application in the identification of epilepsy-related dFC states using simultaneous EEG-fMRI data. With this *l*_1_-DL approach, we aim to accommodate a potentially varying degree of sparsity upon the interchange between epileptic and non-epileptic dFC states. The simultaneous recording of the EEG is used to extract time courses representative of epileptic activity, which are incorporated into the fMRI dFC state analysis to inform the selection of epilepsy-related dFC states. We found that the proposed *l*_1_-DL method performed best at identifying epilepsy-related dFC states, when compared with two alternative methods of extreme sparsity (*k*-means clustering, maximum; and principal component analysis, minimum), as well as an *l*_0_-norm regularization framework (*l*_0_-DL), with a fixed amount of temporal sparsity. We further showed that epilepsy-related dFC states provide novel insights into the dynamics of epileptic networks, which go beyond the information provided by more conventional EEG-correlated fMRI analysis, and which were concordant with the clinical profile of each patient. In addition to its application in epilepsy, our study provides a new dFC state identification method of potential relevance for studying brain functional connectivity dynamics in general.

## Introduction

The simultaneous acquisition of the electroencephalogram (EEG) and blood-oxygen-level-dependent (BOLD) functional magnetic resonance imaging (fMRI) has been playing a major role in better understanding human brain function in general, but particularly in the field of epilepsy. In fact, while EEG can sample the relatively fast electrical oscillations abnormally generated by hypersynchronized populations of neurons, the remarkable spatial resolution of fMRI enables their accurate localization in the brain^1–4^. Typically, features representative of epileptic activity are extracted from the EEG data and brain regions exhibiting correlated BOLD signal changes are identified as the epileptic network^5–8^. This is especially important in the case of patients with drug-refractory focal epilepsy underdoing evaluation for surgical treatment^9–13^. With this approach, brain regions within the epileptic network are assumed to be functionally connected in a static fashion. More generally, several networks of functionally connected brain regions can be found in the normal brain during rest, the so-called resting-state networks (RSNs), which exhibit temporally correlated spontaneous fluctuations in the BOLD signal, referred to as functional connectivity (FC)^14^.

Although most studies of brain FC have assumed temporal stationarity, there is increasing evidence that brain networks are in fact continuously reorganizing in response to both internal and external stimuli, resulting in temporal fluctuations of FC within and between networks across multiple time-scales^15,16^. This is especially important in epilepsy, which is characterized by the interchange between epileptic and normal brain states associated with the spontaneous occurrence of epileptic activity^17^. A growing field of research is now dedicated to the study of dynamic functional connectivity (dFC), as reviewed in^15,18^. By far the most common strategy to study dFC is based on first parcellating the brain into a number of brain regions and then employing a sliding window approach to compute the Pearson correlation between each pair of brain regions over a number of overlapping time windows^19,20^. Interestingly, reoccurring dFC spatial patterns, or states, have been found across time and subjects by employing unsupervised machine learning techniques, in particular *k*-means clustering and principal component analysis (PCA)^20,21^. While clustering assumes that, at each time instant, dFC is described by a single dFC state, PCA describes dFC as a linear combination of all dFC states with different weights over time. One study compared these two methods and their underlying assumptions regarding the temporal sparsity of dFC on both task and resting-state fMRI data, by proposing the use of an *l*_0_-norm regularized dictionary learning approach^22^. Briefly, dictionary learning is a matrix factorization method that describes each dFC matrix as a linear combination of a subset of dFC states, thus imposing a certain degree of temporal sparsity. When using an *l*_0_-norm regularization framework, the number of states comprising the subset is determined by a hyperparameter and fixed over time. In contrast, with *l*_1_-norm regularization, the number of elements in the subset is allowed to vary over time. Although dictionary learning allows for variable constraints in the temporal domain, intermediate degrees of sparsity have not yet been tested.

Despite the increasing literature on methods for estimating brain states from BOLD dFC data, the neurophysiological underpinnings of dFC fluctuations, and consequently brain states, are still not fully understood. Given that the dFC fluctuations under study occur spontaneously, the identification of their electrophysiological correlates requires the simultaneous acquisition of EEG data, and a number of studies have previously followed this approach^23^. One first study investigated if fluctuations in EEG power within the alpha and theta bands, which are known to reflect states of arousal, were related with changes in connectivity between the task-negative default mode network (DMN) and the task-positive dorsal attention network (DAN). The authors found a positive/negative correlation between the alpha/theta EEG power and the DMN-DAN connectivity over time, respectively. Their finding is in agreement with other studies reporting that decreased connectivity between DMN and task-positive networks reflects high arousal periods^19^. In the context of epilepsy, standard EEG-correlated fMRI analyses have provided crucial insights into the brain areas involved in the generation of epileptic activity^2–6,8,24^. Motivated by this observation and the categorization of epilepsy as a network disease^25^, a few studies have integrated simultaneously recorded EEG information onto the dFC analyses in order to identify network connectivity changes associated with epileptic activity^17,26–28^. Apart from two studies^17,28^, the information retrieved from the EEG data has typically only been used to guide the dFC analysis rather than being fully integrated in the dFC estimation^26,27^.

In this work, we propose a novel methodology for the identification of epilepsy-related dFC states from simultaneous EEG-fMRI data, consisting of: (1) estimation of dFC states using an *l*_1_-norm regularized dictionary learning (*l*_1_-DL) approach, allowing for an intermediate, and variable, degree of sparsity in time; and (2) identification of epilepsy-related dFC states by incorporating information from concurrent EEG recordings. A group of eight focal epilepsy patients was selected for the purpose of testing the proposed methodology, and comparing it with two factorization methods of extreme sparsity: *k*-means clustering (*k*-CL; maximum) and PCA (minimum). Extending the work by^22^, and applying it in the context of epilepsy, intermediate, but fixed, degrees of sparsity were also tested by means of an *l*_0_-norm regularized dictionary learning (*l*_0_-DL) approach.

## Methods

**Figure 1:**
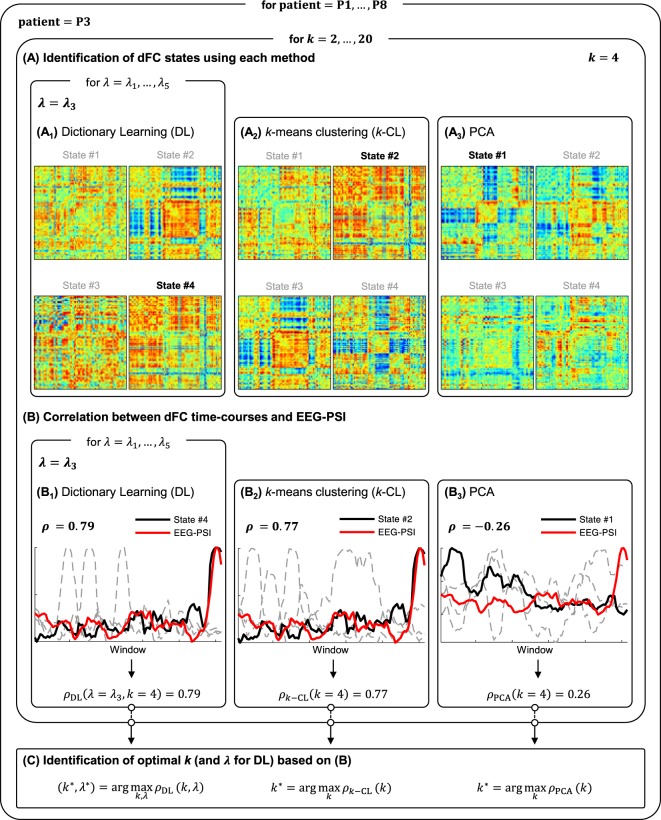
*Schematic diagram of the processing pipeline (example from patient P3)*. After the estimation of dFC using a sliding-window Pearson correlation approach: (**A**) dFC states are identified using DL (A_1_), *k*-means clustering (A_2_) and PCA (A_3_), ranging the number of states *k* to be estimated, and the regularization parameters λ in DL. The dFC states estimated using each method for *k* = 4 are shown. (**B**) The respective non-sparse weight time-courses (dashed gray or black traces) are correlated with the EEG-PSI metric (red trace, representative of the epileptic activity), and the state yielding the highest correlation *ρ* is identified. The optimal *k*, and λ for DL, are determined for each patient and method as those that maximize *ρ*.

Data acquisition procedures will be presented first, followed by the methods employed to estimate dFC and to integrate EEG-based metrics representative of epilepsy-related BOLD signal changes in the subsequent identification of dFC states. The main steps of the proposed methodology are depicted in Fig. 1. All the procedures described next were coded in MATLAB®, and are available together with all data upon request.

### Patient selection

The current population cohort comprises fourteen drug-refractory focal epilepsy patients. These were recruited by the clinical team from the Program of Surgery for Epilepsy of the Hospital Center of West Lisbon, and underwent an EEG-fMRI study at the Imaging Center of Hospital da Luz in Lisbon, Portugal. All adult patients gave written informed consent; a parent and/or legal guardian provided the informed consent in the cases of patients under 18 years old. This study was designed in accordance with the Declaration of Helsinki. The methods carried out in this study, namely those regarding patient recruitment, experimental protocols and data acquisition, were evaluated and approved by the Ethics Committee of Hospital Center of West Lisbon (Lisbon, Portugal). Because of the known effects of anesthesia regarding the suppression of BOLD fluctuations of neuronal original^29^, seven patients who were submitted to sedation during their MRI examination following local guidelines for small children and uncooperative patients were excluded, and thus only eight patients were eligible for the current study. According to the visual inspection by the neurophysiologist in the team, epileptic activity was recorded on the EEG of five patients (P1, P2, P3, P4 and P5) during the EEG-fMRI study: an epileptic seizure was recorded on patient P3, and inter-ictal epileptiform discharges (IEDs) were recorded on patients P1, P2, P4 and P5. No epileptic events were detected on the EEG of the remaining three patients (P6, P7 and P8). Details of all patients are provided in Table 1.

**Table 1:** *Characterization of the EEG-fMRI datasets for all patients.*

Patient	Age [years]	Dataset	Duration [min]	# IEDs	Mean head displacement [mm]	Clinical Condition
P1	8	1*	10	387	N.A.	CSWS^1^, with right neonatal thalamic hemorrhage and IEDs over the posterior right quadrant epileptogenic focus and frontal propagation.
		2	20	754	0.26	
		3	10	292	0.12	
P2	9	1*	10	164	N.A.	CSWS^1^, with IEDs over the left temporal lobe, and verbal agnosia (Wernicke type) and impaired ability to sustain attention.
		2	10	596	0.04	
		3	20	738	0.06	
P3	11	1*	10	—	N.A.	Childhood absence epilepsy (CAE), with IEDs restricted to the left hemisphere.
		2	10	1 (seizure)	0.14	
P4	27	1*	10	70	N.A.	Refractory focal epilepsy, with IEDs over the posterior occipital-temporal lobe, and frontal propagation.
		2	10	15	0.19	
		3	20	7	0.21	
P5	33	1*	10	837	N.A.	Continuous partial epilepsy, with large left-temporal cortical dysplasia, accompanied by continuous myoclonias of the right hand.
		2	10	288	0.16	
		3	10	287	0.12	
		4	10	342	0.15	
P6^†^	15	1*	10	0	N.A.	Benign occipital epilepsy, with IEDs prominently over the left hemisphere.
		2	5	0	0.18	
		3	20	0	0.11	
		4	10	0	0.13	
P7^†^	15	1*	10	0	N.A.	IEDs over the frontal lobe bilaterally, with a hypothesized hypothalamic hamartoma.
		2	10	0	0.23	
		3	10	0	0.19	
P8^†^	16	1*	10	0	N.A.	IEDs over the frontal lobe and a poorly characterized hyper-intense region on structural MR images, compatible with an hypothalamic hamartoma.
		2	10	0	0.12	
		3	10	0	0.10	

The duration of the datasets, the number of IEDs/seizures identified in each case, and the mean head displacement are reported, the latter estimated by the FSL’s motion correction tool (MCFLIRT^39^). A brief description of each patient’s clinical picture at the time of the simultaneous EEG-fMRI studies is also provided. The EEG datasets acquired outside the MR scanner are indicated by the*. The patients with no clear epileptic activity recorded on the EEG are indicated by^†^.

### EEG-fMRI data acquisition

“Imaging was performed on a 3 Tesla Siemens Verio scanner (Siemens, Erlangen) using a 12-channel RF receive coil. Functional images were acquired using a 2D multi-slice gradient-echo echo-planar imaging (EPI) sequence, with TR/TE = 2500/30 ms, from 37 or 40 contiguous axial slices with interleaved acquisition, and 3.5 × 3.5 × 3.0 mm^3^ voxel size, yielding whole-brain coverage. Whole-brain, 1 mm isotropic structural images were acquired using a T_1_-weighted 3D gradient-echo MPRAGE sequence. EEG data were recorded using an MR-compatible 32-channel BrainAmp MR plus amplifier (Brain Products, Germany). A standard BrainCap MR model (EasyCap, Herrsching, Germany) was used, containing 31 Ag/AgCl ring-type electrodes arranged according to the 10–20 system, a dedicated electrode for referencing, and one electrode placed on the back for electrocardiogram (ECG) recording. Sampling was performed at 5000 Hz, synchronized with the scanner’s 10 MHz clock. For each patient, two or three simultaneous EEG-fMRI runs of 5, 10 or 20 min were then performed inside the MR scanner, yielding a total recording time of 30 min. Overall, 10/7 simultaneous EEG-fMRI datasets were acquired from the 5/3 selected patients with/without clear epileptic activity recorded on the EEG, respectively”^8^.

### EEG data analysis

#### Pre-processing steps

EEG datasets from all patients underwent gradient artifact correction on a volume-wise basis using a standard artifact template subtraction (AAS) approach^30^. For the removal of the pulse artifact, the method presented in^31^ was employed, whereby the EEG data is first decomposed using independent component analysis (ICA), followed by AAS to remove the artifact occurrences from the independent components (ICs) associated with the artifact. The corrected EEG data is then obtained by reconstructing the signal using the artifact-corrected ICs together with the original non-artifact-related ICs. Finally, the EEG data was down-sampled to 250 Hz and band-pass filtered to 1–45 Hz. For the purpose of removing the pulse artifact from the EEG, and the physiological noise from the fMRI (see below), the Pan-Tompkins algorithm^32^ was optimized and used for the detection of R peaks on the ECG data^33^.

#### EEG predictor of epilepsy-related BOLD-fMRI fluctuations

In order to identify epilepsy-related BOLD dFC changes, fluctuations representative of epileptic activity were extracted from the EEG for all patients resorting to synchronization measures, particularly the phase synchronization index (PSI). This feature was chosen because we have recently shown that it yields a more sensitive and specific predictor of the associated BOLD signal when compared to other commonly used univariate EEG metrics, irrespective of the presence of epileptic activity on the EEG^8^. PSI is a measure of the phase difference between the EEG signals recorded in two different channels. Because the common reference and volume conduction of the source activity in scalp recordings are known to overestimate synchronization measures, these were tackled by applying the Surface Laplacian transformation to the EEG data as implemented in the MATLAB® toolbox SSLTool (http://ssltool.sourceforge.net/). From the resulting current density data, the spectral profile of each channel was obtained by time-frequency decomposition using Morlet wavelets^34,35^. PSI was then computed, for each pair of channels *l* and *m*, and for each time segment *j* and frequency bin *f*, according to the equation^36^:

1 $$PS{I}_{lm}(j,f)=\sqrt{{X}_{lm}{(j,f)}^{2}+{Y}_{lm}{(j,f)}^{2}}$$

with

2 $${X}_{lm}(j,\,f)=\frac{1}{N}\sum _{t=(j-1)N+1}^{jN}\,\cos ({\rm{\Delta }}{\phi }_{lm}(t,f))\,{\rm{and}}\,{Y}_{lm}=\frac{1}{N}\sum _{t=(j-1)N+1}^{jN}\,\sin ({\rm{\Delta }}{\phi }_{lm}(t,f))$$

where ${\rm{\Delta }}{\phi }_{lm}(t,f)$ is the instantaneous phase difference between the EEG signals from channels *l* and *m* at time point *t* and frequency bin *f*; and *N* is the number of time points within segment *j*. In order to obtain one PSI value per channel pair, frequency bin and fMRI volume, we defined each segment with a duration of 2.5 s, matching the repetition time of the fMRI acquisition. For each patient, a single channel pair was selected as the one yielding the PSI with the highest temporal variance. The PSI of this selected channel pair was then averaged across the frequency band 3–10 Hz, in order to more specifically encompass the spectral profiles of the epileptic activity recorded on patients P1-P5. A single PSI time course was thus obtained for each patient, yielding the EEG-PSI metric. Further details regarding the extraction of EEG-PSI can be found in^8^.

### MRI data analysis

#### Data pre-processing

“The following pre-processing steps were applied to the fMRI data recorded from all patients prior to subsequent analyses. The first three volumes were discarded to allow the signal to reach the steady-state, and non-brain tissue was removed using FSL’s tool BET^37^. Cardiac and respiratory traces synchronized with the fMRI acquisition were obtained from the ECG recording following the methodology in^33^. Quasi-periodic BOLD fluctuations related to cardiac and respiratory cycles were modeled by a fourth order Fourier series using RETROICOR^38^ and removed by linear regression. Subsequently, slice timing and motion correction were performed using FSL’s tool MCFLIRT^39^. Next, aperiodic BOLD fluctuations associated with changes in the heart rate as well as in the depth and rate of respiration were modeled by convolution with the respective impulsive response functions (as described in^40^) and removed by linear regression. Additionally, the average BOLD fluctuations in white matter (WM) and cerebrospinal fluid (CSF), as well as the six motion parameters (MPs) estimated by MCFLIRT, were also regressed out of the data as confounds. Finally, high-pass temporal filtering with a cut-off period of 100 s and spatial smoothing using a Gaussian kernel with full width at half-maximum (FWHM) of 5 mm were performed.

For each patient, WM and CSF masks were obtained from the respective T_1_-weighted structural image by segmentation into gray matter, WM and CSF using FSL’s tool FAST^41^. The functional images were co-registered with the respective T_1_-weighted structural images and the Montreal Neurological Institute (MNI)^42^ template, using FSL’s tool FLIRT^39,43^. Both WM and CSF masks were transformed into functional space and then eroded using a 3 mm spherical kernel in order to minimize partial volume effects^44^. Additionally, the eroded CSF mask was intersected with a mask of the large ventricles from the MNI space, following the rationale described in^8,45^.

Each patient’s structural image was parceled into *R* = 90 non-overlapping regions of interest (ROIs) according to the automated anatomical labeling (AAL) atlas^46^. These ROIs were co-registered to the patient’s functional space, and the pre-processed BOLD data were then averaged within each ROI. The resulting signals were low-pass filtered with a cutoff frequency of 0.1 Hz, because synchronized BOLD fluctuations of neuronal origin are mainly reflected within this frequency range^14,47^.

#### dFC state identification

For all patients, dFC was first estimated by means of a sliding window approach using a window length of 37.5 s (15 TRs) and a step size of 5 s (2 TRs). The pairwise Pearson correlation coefficient was computed for all pairs of parcel-averaged BOLD signals, and for each sliding window. The final dFC matrix was obtained by extracting the upper triangular part of each correlation matrix and vectorising it for all windows, yielding a matrix ${\bf{C}}=[{{\bf{c}}}_{1},\ldots ,{{\bf{c}}}_{T}]\in {{\mathbb{R}}}^{M\times T}$, where $M=({R}^{2}-R)/2$ and *T* denotes the number of windows. The identification of dFC states was then performed using the following four methods.

The proposed *l*_1_-norm regularized DL method can be formulated as the following matrix factorization problem:

3 $${\bf{C}}={\bf{D}}{\bf{A}}$$

where ${\bf{D}}=[{{\bf{d}}}_{1},\ldots ,{{\bf{d}}}_{k}]\in {{\mathbb{R}}}^{M\times k}$ and ${\bf{A}}=[{{\bf{a}}}_{1},\ldots ,{{\bf{a}}}_{T}]\in {{\mathbb{R}}}^{k\times T}$ represent the dFC states and associated weight time-courses, respectively; and *k* is the number of dFC states. The identification of the dFC states **D** can then be formulated as an optimization problem given by:

4 $$\text{arg}\mathop{\min }\limits_{{\rm{D}},{\rm{A}}}{\Vert {\bf{C}}-{\bf{D}}{\bf{A}}\Vert }_{F}^{2}$$

where **D** and **A** are estimated so that the reconstruction error of **C**, $E={\Vert {\bf{C}}-{\bf{D}}{\bf{A}}\Vert }_{F}^{2}$, is minimized; ${\Vert \cdot \Vert }_{F}$ denotes the Frobenius norm of a matrix.

The estimation of **D** and **A** was performed using the algorithms implemented in the MATLAB® toolbox SPArse Modeling Software (SPAMS^48^). Since the problem formulated in Eq. (4) is not jointly convex in **D** and **A**, an iterative estimation procedure is used, whereby these matrices are estimated separately, by keeping one of them fixed. Specifically, the optimization problems of estimating **D** and **A**, respectively, were formulated as:

5 $${\rm{\arg }}\mathop{{\rm{\min }}}\limits_{{\rm{D}}}\frac{1}{T}\sum _{i=1}^{T}\frac{1}{2}{\Vert {{\bf{c}}}_{i}-{\bf{D}}{{\bf{a}}}_{i}\Vert }_{2}^{2}+\lambda {\Vert {{\bf{a}}}_{i}\Vert }_{1}$$

6 $$\text{arg}\mathop{min}\limits_{{\rm{A}}}{\parallel {\bf{C}}-{\bf{D}}{\bf{A}}\parallel }_{2}^{2}{\rm{s}}.{\rm{t}}.{\parallel {\bf{A}}\parallel }_{1} < \lambda $$

where *λ* is the non-negative parameter that controls the degree of sparsity of the solutions on an *l*_1_-norm regularization framework; **c**_***i***_ and **a**_*i*_ denote the *i*^th^ column and row of **C** and **A**, respectively. In this work, we ranged the number of dictionaries, *k*, from 2 to 20 in unitary steps; the regularization parameter, *λ*, was also ranged within five values determined empirically and specifically for each patient, in order to guarantee solutions of intermediate sparsity in all cases. In its original formulation, the first iteration on Eq. (4) requires that the dictionaries **D** are randomly initialized^48^. Here, we tested such implementation, but also investigated the impact of initializing the optimization procedure with dictionaries estimated through *k*-CL and PCA, as described next.

For comparison purposes, *k*-CL, PCA and *l*_0_-DL were tested; these are also techniques of matrix factorization, derived by adding specific constraints and/or regularizations to Eq. (4). For *k*-CL, the MATLAB® implementation of the *k*-means algorithm was used, varying the number of clusters *k* from 2 to 20 with unitary step, the same used in DL. For each *k* value, the procedure was repeated 100 times with random initializations in order to avoid the convergence to local minima. The *l*_1_-norm was used to compute the distance matrix used for *k*-CL, as recommended in^20^. In this case, each cluster represents a dFC state, and is characterized by a connectivity matrix **D** (its centroid), as well as a series of all-or-none occurrences **A**. For PCA, the MATLAB® implementation was used to estimate *k* = 20 PCs, to match the highest number of clusters and dictionaries tested for *k*-CL and the DL approaches, respectively. Each PC represents a dFC state, and is characterized by a connectivity matrix **D** (the eigenvector), as well as a non-sparse weight time-course **A** (computed by projecting the dFC matrix onto the respective principal component).

Finally, the estimation of **D** and **A** by *l*_0_-DL was also performed using the SPAMS toolbox^48^. The respective optimization problem can be simply formulated by replacing the *l*_1_-norm in Eqs (5) and (6) by the *l*_0_-norm. The number of dictionaries was ranged from 3 to 20 in unitary steps. In this case, the regularization parameter determines the number of nonzero entries in each column of **A** (**a**_***i***_), and it was ranged from 2 to *k*−1, with an upper limit of 6 (approximately matching the highest number of nonzero entries observed when using the range of *λ* values for *l*_1_-DL). In this way, the extreme cases of sparsity are excluded (since *λ* = 1 and *λ* = *k* reflect approximately the already considered cases of *k*-CL and PCA, respectively). As for *l*_1_-DL, the impact of initializing the optimization procedure with dictionaries estimated through *k*-CL and PCA was also investigated when using *l*_0_-DL.

#### Optimization and comparison of dFC state identification methods

All dFC state identification methods described above were optimized and compared based on their ability to retrieve epilepsy-related dFC states among the identified dFC states. Because our previous study showed that EEG-PSI is an accurate proxy of epilepsy-related EEG fluctuations, such ability was quantified by comparing the contribution of each dFC state over time with the EEG-PSI metric. In contrast with PCA, the contributions estimated by *k*-CL and the DL approaches are sparse in time. For consistency purposes, non-sparse weight time-courses were computed for all methods by correlating each dFC state’s matrix **D** with the dFC matrix **C** of each time window. The correlation coefficient, *ρ*, was computed between the non-sparse weight time-course of each dFC state and the EEG-PSI metric, for each patient and method, and for each value of *k*, and also each value of *λ* in the case of the DL approaches. For each patient and method, the values of *k* (and *λ* in the DL approaches) yielding the maximum *ρ* (*ρ*_max_) were identified; for PCA, the absolute values were considered since the sign of the states is arbitrary in this case. Because we found that, in some cases, increasing *k* beyond a certain value only led to marginal increases in *ρ* (see Results section), the optimal number of states was determined as the minimum *k* necessary to achieve 90% of *ρ*_max_; the corresponding *ρ* value (${\rho }_{{\rm{\max }}}^{\ast }$) was considered instead of *ρ*_max_, thus favoring a more parsimonious estimation of dFC states.

For the comparison between methods, the average ${\rho }_{{\rm{\max }}}^{\ast }$ across the datasets of all patients (${\bar{\rho }}_{{\rm{\max }}}^{\ast }$) was computed. In order to further corroborate this comparison criterion, the spatial correlation was computed between the connectivity matrix of the dFC state with ${\rho }_{{\rm{\max }}}^{\ast }$ (dFC_max_) and the average **C** across the time windows in which the EEG-PSI metric reached a certain percentage of its maximum value (*ρ*_dFC_), for each patient and method. The rationale is that such windows correspond to periods of particularly intense epileptic activity as measured by EEG-PSI, and thus are hypothesized to be representative of the epileptic activity of each patient. This percentage was set between 90 and 95% and was determined for each patient such that the number of windows was approximately the same for all patients. For control purposes, the spatial correlation was also computed between dFC_max_ and the average **C** across windows in which the EEG-PSI metric was below 110% of its minimum value (*ρ*_CTRL_). Differences between the performance of the various dFC state estimation methods tested here was evaluated by means of a 1-way repeated measures analysis of variance (ANOVA), for each performance measure separately (i.e, ${\bar{\rho }}_{{\rm{\max }}}^{\ast }$, ${\bar{\rho }}_{{\rm{d}}{\rm{F}}{\rm{C}}}$ and ${\bar{\rho }}_{{\rm{C}}{\rm{T}}{\rm{R}}{\rm{L}}}$). A level of significance *p* < 0.05 was considered.

In order to assess the impact of the window length on the dFC analyses and the performance of the different methods tested, we repeated the procedures described above with four different values of the window length, which have previously been indicated as adequate for dFC estimation^18^: 30.0 s (12 TRs), 45.0 s (18 TRs), 52.5 s (21 TRs) and 60.0 s (24 TRs). First, we computed the spatial correlation between dFC_max_ obtained with the original window length of 37.5 s (15 TRs) and those obtained with the four additional window lengths, in order to assess how stable the dFC states highly correlated with EEG-PSI were across different window lengths. Secondly, we compared the performance measures (average ${\rho }_{{\rm{\max }}}^{\ast }$, ${\rho }_{{\rm{dFC}}}$ and ${\rho }_{{\rm{CTRL}}}$) for all methods and window lengths tested, in order to evaluate the impact of the window length on the performance of each method.

The proposed *l*_1_-DL method with initialization based on dFC states estimated through PCA (*l*_1_-DL_PCA_) yielded the highest ${\bar{\rho }}_{{\rm{\max }}}^{\ast }$ (as presented in the Results and Table 2) and was thus the chosen factorization method, and the one kept for subsequent analyses.

**Table 2:** *Average performance of each dFC state identification method*: correlation coefficients of interest (${\bar{\rho }}_{{\rm{\max }}}^{\ast }$, ${\bar{\rho }}_{{\rm{d}}{\rm{F}}{\rm{C}}}$, ${\bar{\rho }}_{{\rm{CTRL}}}$), averaged separately across patients with (P1-P5) and without (P6-P8) clear epileptic activity.

Method	Performance measure					
	*Patients P1-P5*			*Patients P6-P8*		
	${\bar{\rho }}_{{\rm{\max }}}^{\ast }$	${\bar{\rho }}_{{\rm{d}}{\rm{F}}{\rm{C}}}$	${\bar{\rho }}_{{\rm{CTRL}}}$	${\bar{\rho }}_{{\rm{\max }}}^{\ast }$	${\bar{\rho }}_{{\rm{d}}{\rm{F}}{\rm{C}}}$	${\bar{\rho }}_{{\rm{CTRL}}}$
*k*-CL	0.49	0.61	−0.21	0.37	0.52	−0.25
PCA	0.25	0.44	−0.13	0.19	0.31	−0.15
*l*_0_-DL	0.49	0.50	−0.29	0.30	0.28	−0.38
*l*_0_-DL_CL_	0.52	0.53	−0.34	**0.38**	0.26	−0.47
*l*_0_-DL_PCA_	0.34	0.39	−0.23	0.34	0.18	−0.26
*l*_1_-DL	0.51	0.62	−0.27	0.35	0.54	−0.31
*l*_1_-DL_CL_	0.51	0.60	−0.30	**0.38**	0.23	−0.34
***l***_**1**_ **-DL**_**PCA**_	**0.53**	**0.67**	−0.25	**0.38**	**0.54**	−0.31

The highest values for each correlation coefficient are highlighted in bold. *l*_1_-DL_PCA_ consistently yielded the highest ${\bar{\rho }}_{{\rm{\max }}}^{\ast }$ and ${\bar{\rho }}_{{\rm{d}}{\rm{F}}{\rm{C}}}$ for patients P1-P5, thus being deemed the best method; the same was subsequently observed for patients P6-P8. Overall, PCA was clearly outperformed by *k-*CL and the DL approaches, the latter slightly surpassing *k*-CL.

#### Selection and characterization of epileptic dFC states

First, the statistical significance of the dFC states estimated through *l*_1_-DL_PCA_ was evaluated without imposing any statistical model, by means of a null distribution generated from the data itself for each patient, following the work described in^21^. For that purpose, the **C** matrix of each patient was first FFT transformed and, for each connectivity pair, a random, uniformly distributed, phase was added; the inverse FFT was then applied to transform the data back to the time domain. Next, the patient-specific optimal parameters found for *l*_1_-DL_PCA_ were used to estimate **D** from the phase-randomized **C** matrix. This procedure was repeated 100 times in order to obtain the null distribution. Since a null distribution must be generated for each dFC state, and because the order of the estimated states by *l*_1_-DL_PCA_ is random, it is not possible to obtain a direct association between the estimated **D** from real data and that estimated from phase-randomized data. This was overcome by first computing the reconstruction error *E* of **C** when using each dictionary **d**_i_ and respective time-course **a**_i_ individually, estimated from real and phase-randomized data, according to:

7 $$E(i)={\Vert {\bf{C}}-{{\bf{d}}}_{i}{{\bf{a}}}_{i}\Vert }_{F}^{2}$$

The association was then obtained after sorting the *E* values computed for each simulation run and for the real data. The sorted *E* values from all simulation runs comprise the null distributions against which the sorted *E* values from the real data will be tested. The fifth percentile of the null distribution for each dFC state was then determined, and the states estimated from real data with a reconstruction error below that value were deemed meaningful. Among the statistically meaningful dFC states, those that exhibited a significant (*p* < 0.05, Bonferroni corrected for multiple comparisons) correlation between EEG-PSI and the respective non-sparse weight time-courses were deemed epilepsy-related.

These epileptic dFC states were then further characterized in terms of changes in the strength of dFC states between brain regions encompassing the epileptic network determined by standard EEG-correlated fMRI analysis. For the purpose of mapping this network, the EEG-PSI metric was first convolved with a canonical, double-gamma hemodynamic response function (HRF)^49^. The resulting regressor and its time derivative were used as explanatory variables in a general linear model (GLM) that was subsequently fitted to the BOLD-fMRI data^50^. The epileptic network was then defined as voxels at which the BOLD signal was significantly (cluster thresholding: voxel *Z* > 2.3, cluster *p* < 0.05) related with the EEG-PSI metric. The resulting epileptic network was then mapped onto the AAL parcellation space, whereby AAL parcels that contained more than 5% of voxels belonging to the epileptic network were selected and used to mask the epileptic dFC states. The AAL brain parcels were grouped into the six main brain regions in this atlas: frontal, limbic, occipital, parietal, subcortical and temporal regions (separately for the left and right hemispheres); the thalamus was further isolated from the remaining subcortical structures given its leading role in the epilepsy of the patients studied here (as described in the Results). The dFC strength between AAL groups belonging to the epileptic network and all AAL groups was then averaged, in order to quantify whole-brain connectivity changes with the epileptic network.

## Results

The performance of the different methods used for identifying epileptic dFC states is first presented. The characterization and plausibility of the dFC states identified with *l*_1_-DL_PCA_ (the best method) are discussed next, for each patient.

### Optimization and comparison of dFC state identification methods

We started by comparing the ability of the different dFC factorization methods tested, with different degrees of sparsity, in identifying epilepsy-related dFC states. Results regarding comparisons between the methods’ performance are shown separately for the two subgroup of patients: with (P1-P5) and without (P6-P8) clear epileptic activity during the EEG-fMRI study. This separation in the two subgroups was motivated by the fact that the former should allow a more reliable verification of epileptic dFC states, and hence a clearer comparison between methods; indeed, differences between methods are more evident for patients P1-P5, when compared to patients P6-P8. The results on the latter subgroup serve to demonstrate the applicability of the methods even in the absence of clear epileptic activity. In Table 2, the average ${\rho }_{max}^{\ast }$, *ρ*_dFC_ and *ρ*_CTRL_ values across the two subgroups of patients (${\bar{\rho }}_{{\rm{\max }}}^{\ast }$, ${\bar{\rho }}_{{\rm{d}}{\rm{F}}{\rm{C}}}$ and ${\bar{\rho }}_{{\rm{CTRL}}}$, respectively) are presented, for *k*-CL, PCA and the *l*_0_-DL and *l*_1_-DL methods, the latter with random, *k*-CL based and PCA-based initializations. Consistently across subgroups of patients, PCA yielded the lowest ${\bar{\rho }}_{{\rm{\max }}}^{\ast }$, suggesting that the orthogonality assumption may not hold for this data. While clustering exhibited slightly lower ${\bar{\rho }}_{{\rm{\max }}}^{\ast }$ when compared with the DL approaches, the three DL variants of both *l*_0_-DL and *l*_1_-DL yielded similar ${\bar{\rho }}_{{\rm{\max }}}^{\ast }$ values. Regarding the ${\bar{\rho }}_{{\rm{d}}{\rm{F}}{\rm{C}}}$ results, *l*_1_-DL methods yielded the highest values compared with all other methods, including the *l*_0_-DL variants. Despite these trends for both ${\bar{\rho }}_{{\rm{\max }}}^{\ast }$ and ${\bar{\rho }}_{{\rm{d}}{\rm{F}}{\rm{C}}}$, a significant effect of the method was found with repeated measures ANOVA for the ${\bar{\rho }}_{{\rm{\max }}}^{\ast }$ measure only. Overall, the *l*_1_-DL_PCA_ was the method yielding the highest ${\bar{\rho }}_{{\rm{\max }}}^{\ast }$ and ${\bar{\rho }}_{{\rm{d}}{\rm{F}}{\rm{C}}}$ values across all patients, and it was thus deemed the best method at identifying epilepsy-related dFC states; the subsequent results will be shown only for this method.

In addition, and in contrast with ${\bar{\rho }}_{{\rm{\max }}}^{\ast }$ and ${\bar{\rho }}_{{\rm{d}}{\rm{F}}{\rm{C}}}$, the average correlation coefficient between epileptic dFC states and the average dFC across windows of low EEG-PSI values (${\bar{\rho }}_{{\rm{CTRL}}}$) was low for all methods. The results of these control tests indicate that the state most related with the occurrence of epileptic activity identified with *l*_1_-DL_PCA_ was similar to the average **C** matrix across windows of high EEG-PSI values, but not the average **C** matrix across windows of low EEG-PSI values, as hypothesized. As expected, the performance measures for patients P6-P8 were consistently lower than those obtained for patients P1-P5. This may in part be explained by the lack of sensitivity of EEG-PSI to capture epilepsy-related fluctuations if no, or only negligible, epileptic activity is occurring.

Regarding the comparison of the different window lengths, the average spatial correlation across the two subgroups of patients between the dFC_max_ state obtained with the original window length (37.5 s, 15 TRs) and those obtained with the four additional window lengths are presented in supplementary Table S1. The selected method *l*_1_-DL_PCA_ yielded the highest spatial correlations in most cases; for the remaining few cases, *l*_1_-DL_PCA_ was only surpassed by other *l*_1_-DL variants. This evidences the good stability of the dFC_max_ state when estimated with *l*_1_-DL approaches, and particularly *l*_1_-DL_PCA_, regardless of the chosen window length. In terms of the performance metrics, presented in supplementary Table S2, *l*_1_-DL_PCA_ was found to outperform the remaining methods in most cases, being only occasionally surpassed by other DL methods, particularly those using an *l*_1_-norm regularization framework, and never by PCA or *k*-CL. This is in agreement with the results for the original window length, and also with those from Table S1. Importantly, the standard deviation of the performance measures across all tested window lengths was always below 0.10 for *l*_1_-DL_PCA_, showing that such values were not critically dependent on the window length.

Concerning the criterion used for selecting the optimal number of states *k* to be estimated, supplementary Fig. S1 illustrates the *ρ* values obtained with *l*_1_-DL_PCA_ for all *k* and patients, and highlights the values corresponding to *ρ*_max_ and ${\rho }_{{\rm{\max }}}^{\ast }$. For patients P1, P2, P3 and P4, a smaller *k* value was found when considering ${\rho }_{{\rm{\max }}}^{\ast }$ (rather than *ρ*_max_), with an average decrease in *ρ*_max_ of only 2% and an increase in ${\bar{\rho }}_{{\rm{d}}{\rm{F}}{\rm{C}}}$ of 6% for *l*_1_-DL_PCA_.

### Selection and characterization of epileptic dFC states

**Figure 2:**
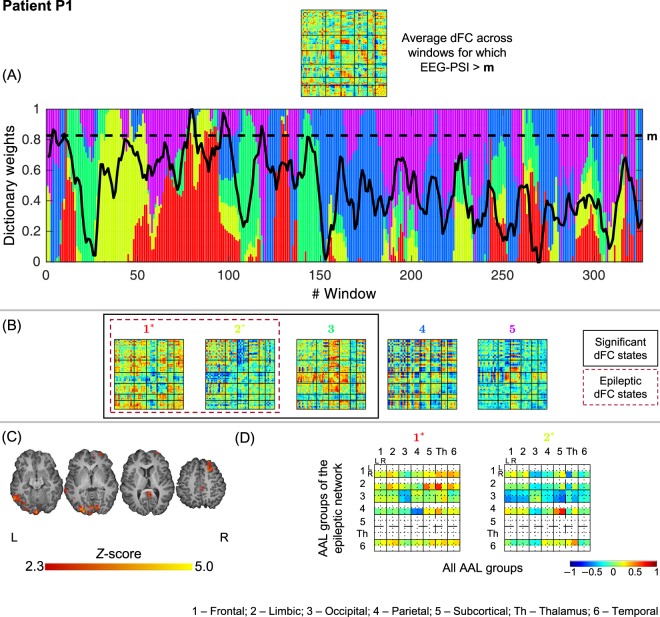
Results of the dFC analyses for patient P1 when using the *l*_1_-DL_PCA_ method. (**A**) Bar plot depicting the contribution (dictionary weight) of the various dFC states (color-coded as in B) over time windows, superimposed with the EEG-PSI metric time course (black trace); the black dashed line represents 90–95% of the EEG-PSI maximum value. All values are normalized between 0 and 1, for visualization purposes. (**B**) Estimated dFC states, ordered by statistical significance and correlation between the respective non-sparse weight time-courses and EEG-PSI (titles are color-coded as in (**A**)). Statistically significant dFC states are highlighted by a solid black square; epilepsy-related dFC states are highlighted by a dashed black square (and indicated with * in the title). All matrices are normalized between −1 and 1. (**C**) Epileptic network obtained by standard EEG-correlated fMRI analysis: *Z* score map of BOLD changes significantly associated with the EEG-PSI, superimposed on the patient’s structural image, shown for four illustrative axial slices. (**D**) Epilepsy-related dFC states with connectivity strengths averaged across the 14 groups of AAL regions (left and right frontal, limbic, occipital, parietal and temporal lobes, thalamus and other subcortical areas). For each group present in the epileptic network shown in (**C**) (vertical axis), the average connectivity strength with all other groups is shown (horizontal axis).

**Figure 3:**
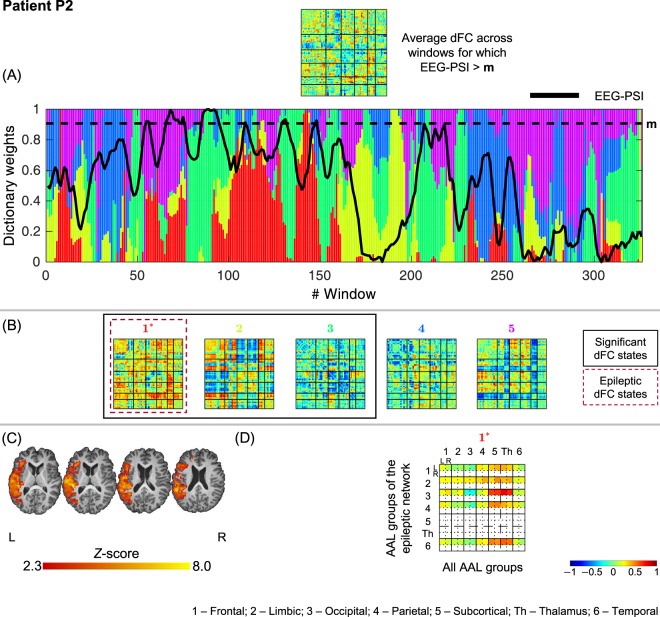
Results of the dFC analyses for patient P2 when using the *l*_1_-DL_PCA_ method.

**Figure 4:**
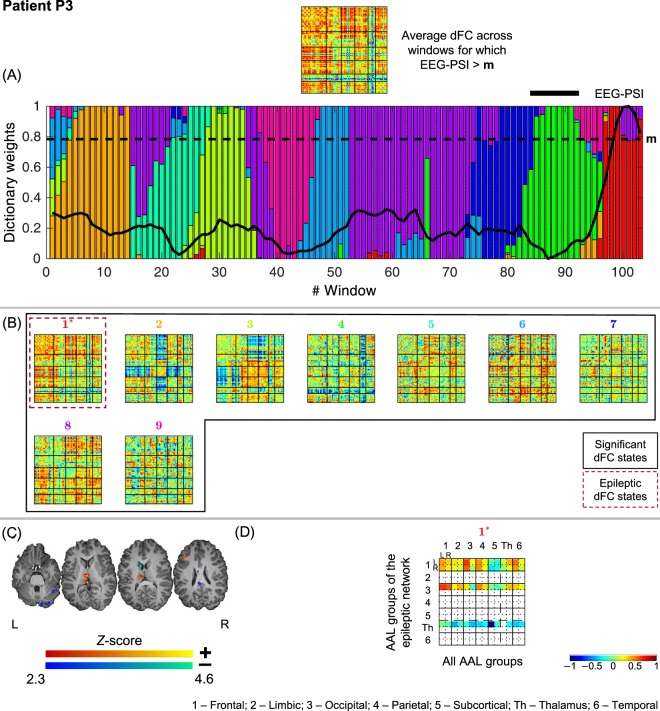
Results of the dFC analyses for patient P3 when using the *l*_1_-DL_PCA_ method.

**Figure 5:**
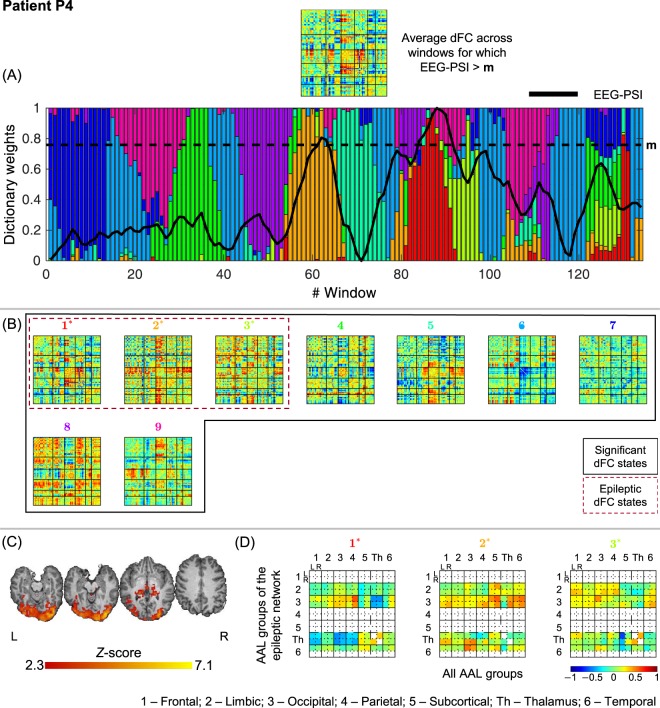
Results of the dFC analyses for patient P4 when using the *l*_1_-DL_PCA_ method.

**Figure 6:**
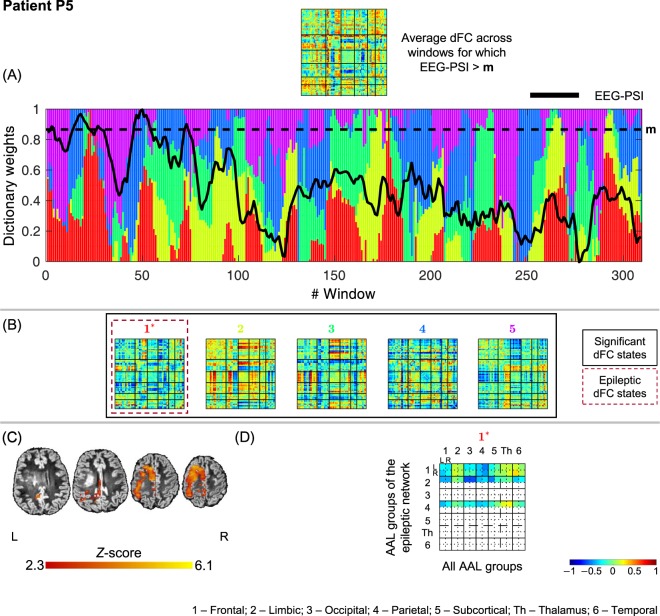
Results of the dFC analyses for patient P5 when using the *l*_1_-DL_PCA_ method.

**Figure 7:**
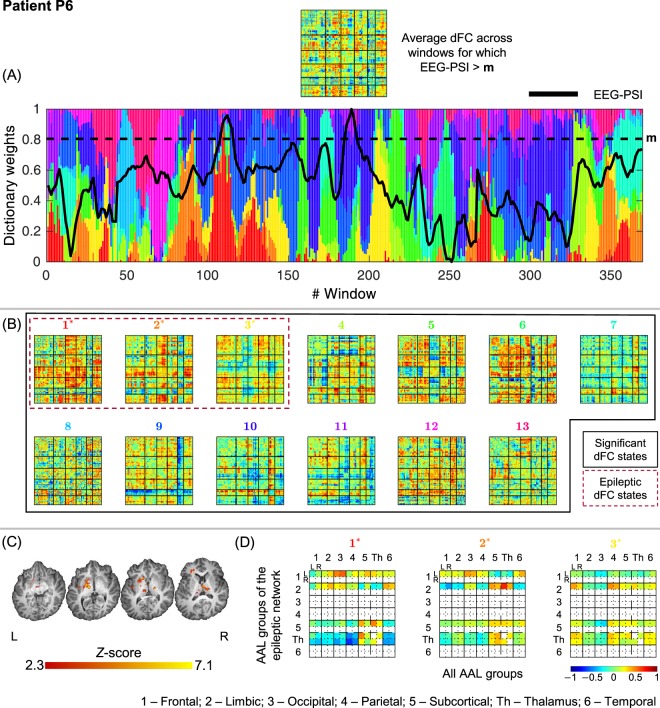
Results of the dFC analyses for patient P6 when using the *l*_1_-DL_PCA_ method.

**Figure 8:**
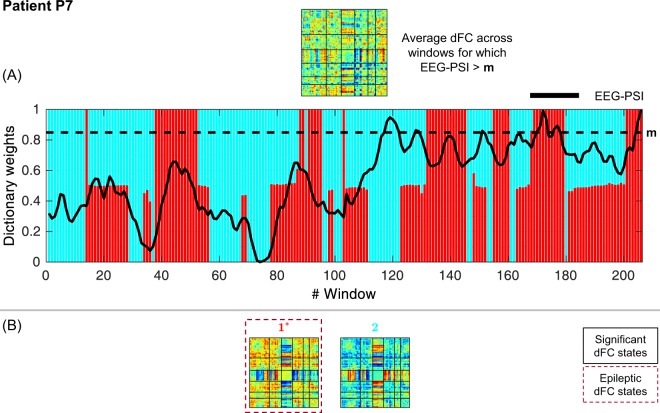
Results of the dFC analyses for patient P7 when using the *l*_1_-DL_PCA_ method.

**Figure 9:**
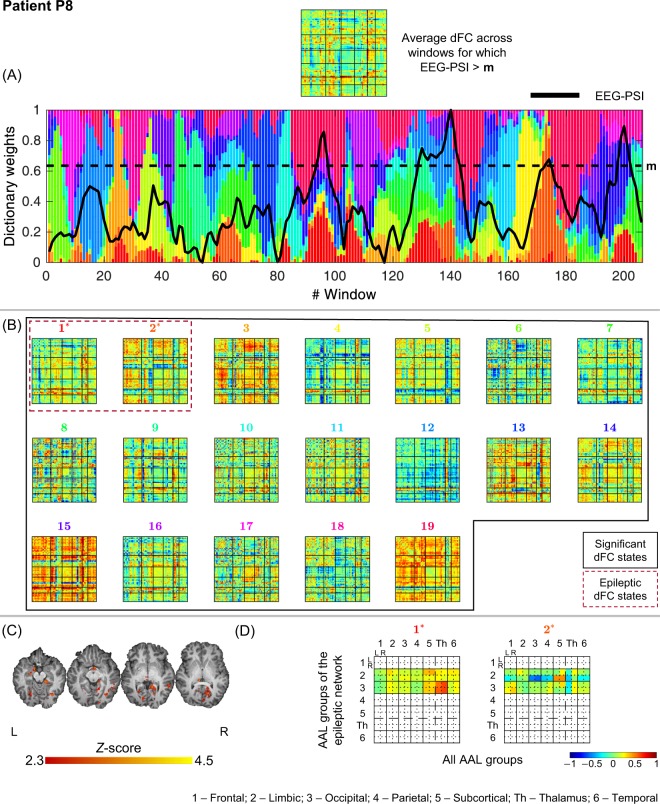
Results of the dFC analyses for patient P8 when using the *l*_1_-DL_PCA_ method.

The results of the dFC analysis leading to the selection and characterization the epileptic dFC states, when using the chosen *l*_1_-DL_PCA_ method, are illustrated in Figs 2–9, respectively for patients P1 to P8. All dFC states are shown, indicating those deemed significant and those deemed epilepsy-related, together with their weights’ time-courses superimposed with the EEG-PSI time-course. The average connectivity strength of epilepsy-related dFC states between AAL groups belonging to the epileptic network and all AAL groups is also presented. Overall, novel insights regarding the dynamics of epileptic activity were provided by the analysis of the epilepsy-related dFC states, which could not be obtained solely based on the epileptic network derived by standard EEG-correlated fMRI analysis. The results obtained in each patient are described in detail below, first for patients with clear epileptic activity (P1-P5) and then for the remaining patients, without clear epileptic activity (P6-P8).

#### P1

3 out of 5 dFC states were statistically meaningful, two of which were deemed epilepsy-related (states #1 and #2). State #1 shows an increased connectivity between right limbic, and left subcortical regions as well as the healthy left thalamus; a decreased connectivity within left parietal regions is also observed. As for state #2, right parietal regions exhibit increased connectivity with subcortical regions, and more strongly with the lesioned right thalamus when compared with the left thalamus. These results support the relationship between the cortical sources of continuous spike-wave of sleep (CSWS) and the early thalamic lesion on the same hemisphere, as postulated in^51^.

#### P2

3 out of 5 dFC states were statistically meaningful, one of which was deemed epilepsy-related (state #1). The most striking findings of this state is the increased connectivity between left occipital, parietal and temporal regions with the thalamus as well as other subcortical regions. The association of the focal CSWS pattern of this patient with a strong connectivity to subcortical areas, and in particular the thalamus, resembles the one seen in patient P1 and further supports the previously proposed link of CSWS and the thalamus^51^.

#### P3

All 9 dFC states identified were statistically meaningful, but only state #1 was deemed epilepsy-related. The very pronounced increase of EEG-PSI values towards the end of the recording is associated with the occurrence of an absence seizure characterized by prolonged bursts of 3 Hz spike and wave complexes circumscribed to the left hemisphere (atypical case of absence epilepsy^52^). During the ascending phase of EEG-PSI preceding the seizure, dFC state #4 dominates and progressively gives way to state #1; the contribution of these states is negligible at windows unrelated with the seizure. The strongly decreased connectivity of the left thalamus with all other brain regions is the most remarkable feature of state #1, and suggests that the positive thalamic BOLD activation found with standard EEG-correlated fMRI analysis, which is well-established in absence seizures^53^, is associated with a decrease in the normally widespread thalamic connectivity. This could also play a major role in the impairment of consciousness associated with the absence seizures.

#### P4

All 9 dFC states identified were statistically meaningful, with states #1, #2 and #3 being deemed epilepsy-related. Two periods of increased epileptic activity were recorded in this patient, as evidenced by the main peaks in the EEG-PSI time-course around windows 60 and 90. For the windows surrounding these peaks, the contribution of states #1 and #2 rose drastically. The most striking observation when analyzing these dFC states in relation with the epileptic network identified by standard EEG-correlated fMRI is the fact that states #1 and #2 exhibit generally increased connectivity across limbic and occipital regions but differ in the thalamic connectivity, which is decreased in #1 and increased in #2. The connectivity between the left thalamus and left subcortical regions is also strongly decreased in state #3.

#### P5

All 5 dFC states identified were statistically meaningful, with state #1 being deemed epilepsy-related. This state exhibits a modest increase in the connectivity of frontal and parietal regions with the thalamus, suggesting that epileptic activity in this cortical dysplasia synchronizes thalamic neurons but not to an extent detectable by the BOLD response in the epileptic network obtained though standard EEG-correlated fMRI analysis. This pattern is also present in P2, but with distinct cortical areas involved in the synchronization process.

#### P6

All 13 dFC states identified were statistically meaningful, with states #1, #2 and #3 deemed epilepsy-related. Despite the absence of clear epileptic activity during the study, the EEG-PSI metric was nevertheless able to detect two peaks at windows 112 and 189, the latter associated with a peak in the contribution of state #1. While state #2 mainly shows increased connectivity between left limbic regions and the left thalamus, increased connectivity between left frontal and occipital regions, as well as the left thalamus and left subcortical regions can be observed for state #1. The increased connectivity between left frontal and occipital regions is consistent with the patient’s semiology, highlighting the involvement of occipital regions, not detected by standard EEG-correlated fMRI analysis.

#### P7

Only 2 dFC states were estimated, and none was found to be statistically meaningful. This result was somewhat expected given the poor mapping of the epileptic network of this patient when using standard EEG-correlated fMRI analysis (also reported in^8^). This may suggest that the absence of epileptic activity in this case precluded the detection of epilepsy-related fluctuations in the EEG-PSI metric.

#### P8

All 19 dFC states identified were statistically meaningful, with states #1 and #2 deemed epilepsy-related. The most striking feature of state #1 is the increased connectivity between occipital and subcortical regions, as well as the thalamus (the latter more pronouncedly). As for state #2, a decreased/increased connectivity between right limbic and occipital/subcortical regions is observed, respectively. These findings are in line with the hypothalamic hamartoma hypothesized for this patient.

## Discussion

We proposed an *l*_1_-norm regularized DL method for the identification of epilepsy-related fMRI dFC states with a flexible degree of temporal sparsity, based on incorporating the highly time-resolved information provided by the EEG recorded simultaneously with fMRI. We systematically compared the ability of DL with two other previously proposed methods with extreme degrees of temporal sparsity, *k*-means clustering and PCA, as well as explored the temporal sparsity of an *l*_0_-norm regularized DL method. We found that the proposed method performed best, especially when using a preliminary PCA for initialization, and further showed that changes in the strength of the epilepsy-related dFC states provide further insights into the dynamics of epileptic networks that go beyond the information provided by more conventional EEG-correlated fMRI analysis.

### Relation with other studies of dFC in epilepsy

A number of studies have previously investigated the dynamics of functional connectivity in epilepsy, using either EEG or fMRI. While EEG-based dFC studies are mainly focused on seizure prediction and characterization^54^ and delineation of epileptogenic foci^55,56^, dFC studies using fMRI mostly aim at identifying dFC features that best discriminate epilepsy patients from healthy controls^57–61^. Only a few studies have attempted to combine the two modalities when analyzing dFC, levering their well-known complementary properties^1–3^. In these studies, dFC is typically measured from the fMRI data, while epilepsy-related information is extracted from the EEG in order to guide the fMRI analyses. In particular, the fMRI signal can be segmented into different epilepsy-related stages based on the EEG (before, during and after the occurrence of an epileptic event, plus a baseline period devoid of epileptic activity). A specific connectivity matrix is computed for each stage, and changes across the successive epilepsy-related stages and in relation to the baseline period are then analyzed^26,27^. For this purpose, long periods in-between epileptic events are necessary in order to define the baseline, which may not be feasible in many cases due to the spontaneous, and often almost continuous, nature of epileptic activity. Only in two reports was the EEG information truly integrated, by comparing an EEG-derived epilepsy-related metric with the dFC time-course computed for each pair of AAL regions, by using Pearson correlation^17^ or wavelet coherence^28^. This yields a single correlation/coherence matrix and the regions specifically associated with epileptic activity are then identified through appropriate thresholding. The major limitation of these approaches lies on the assumption that epileptic activity is described by a single brain network exclusively; in fact, different brain networks have been found at different stages of the epileptic activity of the same patient^26,27^. Taking these considerations into account, a fuzzy clustering approach has also been explored for the identification of epilepsy-related dFC patterns, which was however found to yield only a small degree of fuzziness and therefore performed comparably with *k*-means while outperforming PCA^62^.

### Relation with other methods to identify dFC states

We found that the proposed *l*_1_-norm regularized DL approach was the best at retrieving epileptic dFC states, when compared with *k*-means clustering or PCA. We believe that, by forcing an intermediate, but variable, degree of sparsity in time, a more natural description of the brain’s functional connectivity dynamics is achieved. In fact, while clustering assumes that the brain is at a single state at each given period of time, PCA imposes that all states contribute to the overall brain functional connectivity to some extent. In contrast, with DL, a subset of the estimated states describes the dFC at each time window, the extent of which is limited by the regularization parameter. This feature of DL was particularly relevant when studying patients P3 and P4, for whom clear periods of increased epileptic activity as quantified by EEG-PSI were described by specific dFC states, but more interestingly, the transition to periods of more intense epileptic activity (or even the beginning of a seizure) were described by a gradual replacement of non-epilepsy-related states by epilepsy-related states, and *vice-versa*. While such transition behavior cannot be captured by clustering, PCA does not clearly differentiate the contribution of epilepsy-related states during epileptic periods because all states have a non-null contribution to the overall dFC.

Regarding the *l*_0_-DL and the *l*_1_-DL approaches, their difference lies on the regularization framework: the *l*_0_-norm fixes the number of non-zero entries in the weight matrix (i.e., number of active dFC states) at each time window, whereas the *l*_1_-norm allows for a variable number of active dFC states at each time point. An *l*_0_-DL approach has been previously used for investigating whether dFC patterns are expressed separately or jointly over time^22^. In this work, the authors derived the specific cases of *k*-means and PCA from the general formulation of *l*_0_-DL, by manipulating λ accordingly, and found that *k*-means was the best method at recovering true dFC patterns from data simulating both separate and joint expression of dFC patterns. The authors also found evidence for a separate expression in task-based fMRI data and for joint expression in resting-state fMRI data. Our findings are generally in agreement with this observation, and further extend it to the study of epileptic states. In fact, while in periods of intense epileptic activity a dominant (usually epilepsy-related) dFC state was evident (suggesting separate expression), periods of weak epileptic activity were usually described by a roughly evenly distributed subset of dFC states (suggesting joint expression). Because of the fixed amount of temporal sparsity, *l*_0_-DL was in principle not suitable to analyze the dFC of epileptic patients, which is expected to alternate between periods of separate and joint expressions intimately related with the occurrence of epileptic activity. Our comparison between *l*_1_-DL and *l*_0_-DL approaches corroborated this observation, with *l*_1_-DL generally outperforming *l*_0_-DL in the identification of epileptic dFC states, suggesting that intermediate and variable, rather than fixed, temporal sparsity is necessary to capture dFC changes in epilepsy.

### Relation with other methods to estimate dFC

In this work, we estimated dFC using a standard approach consisting of brain parcellation into 90 AAL regions followed by sliding-window Pearson correlation, according to commonly adopted procedures in the literature. Other sliding-window based strategies have also been used in this context, namely tapered sliding-windows^20^ or exponential weighted moving average^63^. Alternatively, spatial independent component analysis (sICA) has been applied on a sliding-window basis^64^. In order to overcome the selection of a window length, approaches capable of estimating dFC at the quasi-instantaneous level have been proposed, namely: phase coherence connectivity^65,66^ and multiplication of temporal derivatives^67^. Such approaches provide dFC time-series with higher temporal resolution, but also make them more prone to high frequency noise fluctuations^66^. More complex approaches for estimating the dFC can also be found in the literature, namely through wavelet transform coherence^68^, multivariate empirical mode decomposition (MEMD)^69^ and 4D spectral analysis^70^.

Mainly motivated by their simplicity and proved ability to estimate meaningful dFC fluctuations, sliding-window based approaches are still the most common in the literature. Nonetheless, this approach is well-known to present important limitations, mainly associated with the selection of the window length^71^. In fact, different results can be obtained when using different window lengths, which are possibly of physiological relevance^72^. Despite the attempt to draw guidelines for a more informed selection of the window length^71^, there are still no clear general indications regarding which window length is the most suitable for a specific study. Interestingly, the recent review by^18^ revealed that most studies were able to detect meaningful resting-state dFC fluctuations when using a window length between 30 and 60 s. Taking these considerations into account, we opted to also use a conventional sliding-window approach, setting the window length to 37.5 s (15 TRs) and the step size to 5 s (2 TRs), which facilitates the comparison of our results with previous studies. Nonetheless, we tested different window lengths and showed that *l*_1_-DL approaches, and particularly *l*_1_-DL_PCA_, outperform the other methods irrespective of the window length. Moreover, *l*_1_-DL_PCA_ also yielded the most stable epileptic dFC states across different window lengths, which may be the result of a more robust identification.

### Limitations

Statistical inference on the results shown here is not possible at the group level, not only because of the limited number of patients recruited, but also due to the heterogeneity of their epilepsy syndromes. Nonetheless, and despite the lack of a ground truth, the results obtained for each individual patient were not only consistent with the respective clinical profiles and standard EEG-correlated fMRI analyses, but also provided further insights into the dynamics of epileptic networks that go beyond the information provided by such analyses. This was observed for each patient, demonstrating the applicability of the proposed methodology at the individual level and to a range of different types of epilepsy, rather than only limited to one specific syndrome.

### Conclusion

This is the first study to apply a dictionary learning method of intermediate, and variable, sparsity in time for dFC state estimation, and to fully incorporate EEG information for the identification of epilepsy-related dFC states. We compared the dictionary learning method with other methods of extreme sparsity, and found that it was the best suited for this purpose. This finding suggests that functional connectivity in the epileptic brain is best described by a combination of multiple dFC states that changes over time (in terms of the contributing dFC states and their contribution weights). Our results also show that, by analyzing the epilepsy-related dFC states, novel insights can be gained on the changes in functional connectivity across brain networks that underlie the spontaneous occurrence of epileptic activity, with potential impact for the pre-surgical evaluation of these patients. Moreover, the proposed methodology provides a new dFC state identification method of potential relevance for studying brain functional connectivity dynamics in general.

## Supplementary information

Supplementary Information
