# Supplementary material for: Identification of epileptic brain states by dynamic functional connectivity analysis of simultaneous EEG-fMRI: a dictionary learning approach
Source: Sci Rep. 2019 Jan 24;9:638. doi: 10.1038/s41598-018-36976-y (PMC6345787; doi:10.1038/s41598-018-36976-y)
Supplement: Supplementary file 1 — Supplementary Information [file 41598_2018_36976_MOESM1_ESM.pdf]

Title

Identification of epileptic brain states by dynamic functional connectivity analysis of simultaneous EEG-fMRI: a dictionary learning approach

Authors

Rodolfo Abreu[1,*], Alberto Leal[2], Patrícia Figueiredo[1]

Affiliations

[1]ISR-Lisboa/LARSyS and Department of Bioengineering, Instituto Superior Técnico – Universidade de Lisboa, Portugal

[2]Department of Neurophysiology, Centro Hospitalar Psiquiátrico de Lisboa, Lisbon, Portugal

* *Corresponding author*

Email address: rodolfo.abreu@tecnico.ulisboa.pt
Phone number: +351 919 623 032

Method	Window length							
	**12 TRs (30.0 s)**		**18 TRs (45.0 s)**		**21 TRs (52.5 s)**		**24 TRs (60.0 s)**	
	P1-P5	*P6-P8*	*P1-P5*	*P6-P8*	*P1-P5*	*P6-P8*	*P1-P5*	*P6-P8*
k-CL	0.75	0.67	0.77	0.75	0.70	0.66	0.74	0.47
PCA	0.64	0.66	0.45	0.35	0.24	0.33	0.33	0.58
l_0-DL	0.58	0.30	0.67	0.12	0.55	0.27	0.57	0.40
l_0-DL_{CL}	0.68	0.50	0.71	0.58	0.82	0.55	0.77	**0.62**
l_0-DL_{PCA}	0.37	0.37	0.38	0.61	0.28	0.62	0.33	0.64
l_1-DL	0.89	0.54	0.75	**0.90**	0.84	**0.81**	0.84	0.59
l_1-DL_{CL}	**0.95**	0.67	0.84	0.64	0.75	0.49	0.79	0.55
**l_1-DL_{PCA}**	0.81	**0.88**	**0.90**	0.75	**0.91**	0.56	**0.84**	0.56

Table S1: Average spatial correlation across patients from the two subgroups, between dFC_{max} obtained with the original window length of 37.5 s (15 TRs) and those obtained with the four window lengths: 12 TRs (30.0 s), 18 TRs (45.0 s), 21 TRs (52.5 s) and 24 TRs (60.0 s). l_1-DL_{PCA} outperformed all the other methods in most cases, being only surpassed by other l_1-DL variants.

Methods	Performance measures																							
	**Window length: 12 TRs = 30.0 s**						**Window length: 18 TRs = 45.0 s**						**Window length: 21 TRs = 52.5 s**						**Window length: 24 TRs = 60.0 s**					
	Patients P1-P5			*Patients P6-P8*			*Patients P1-P5*			*Patients P6-P8*			*Patients P1-P5*			*Patients P6-P8*			*Patients P1-P5*			*Patients P6-P8*		
	$\bar{\rho}^*_{\max}$	$\bar{\rho}_{\mathrm{dFC}}$	$\bar{\rho}_{\mathrm{CTRL}}$	$\bar{\rho}^*_{\max}$	$\bar{\rho}_{\mathrm{dFC}}$	$\bar{\rho}_{\mathrm{CTRL}}$	$\bar{\rho}^*_{\max}$	$\bar{\rho}_{\mathrm{dFC}}$	$\bar{\rho}_{\mathrm{CTRL}}$	$\bar{\rho}^*_{\max}$	$\bar{\rho}_{\mathrm{dFC}}$	$\bar{\rho}_{\mathrm{CTRL}}$	$\bar{\rho}^*_{\max}$	$\bar{\rho}_{\mathrm{dFC}}$	$\bar{\rho}_{\mathrm{CTRL}}$	$\bar{\rho}^*_{\max}$	$\bar{\rho}_{\mathrm{dFC}}$	$\bar{\rho}_{\mathrm{CTRL}}$	$\bar{\rho}^*_{\max}$	$\bar{\rho}_{\mathrm{dFC}}$	$\bar{\rho}_{\mathrm{CTRL}}$	$\bar{\rho}^*_{\max}$	$\bar{\rho}_{\mathrm{dFC}}$	$\bar{\rho}_{\mathrm{CTRL}}$
k-CL	0.46	0.51	−0.18	0.31	0.57	−0.09	0.52	0.58	−0.30	0.43	0.54	−0.09	0.53	0.60	−0.34	0.45	0.47	−0.09	0.55	0.67	−0.39	0.43	0.52	−0.48
PCA	0.25	0.44	−0.13	0.19	0.31	−0.15	0.31	0.41	−0.17	0.17	0.32	−0.16	0.37	0.48	−0.27	0.25	0.37	−0.20	0.33	0.46	−0.21	0.30	0.42	−0.17
l_0-DL	0.47	0.47	−0.22	0.33	0.22	−0.26	0.56	0.64	−0.40	0.48	0.50	−0.26	**0.58**	0.62	−0.52	0.47	0.54	−0.26	0.56	0.57	−0.50	0.47	0.57	−0.39
l_0-DL$_{\mathrm{CL}}$	**0.48**	0.48	−0.30	0.35	0.36	−0.32	0.54	0.49	−0.41	0.48	0.55	−0.32	0.57	0.60	−0.48	**0.52**	0.51	−0.32	0.59	0.68	−0.54	0.50	0.56	−0.49
l_0-DL$_{\mathrm{PCA}}$	0.38	0.35	−0.26	0.33	0.20	−0.26	0.41	0.44	−0.26	**0.50**	0.53	−0.26	0.41	0.34	−0.42	0.46	0.43	−0.26	0.42	0.37	−0.21	0.47	0.57	−0.40
l_1-DL	0.46	0.56	−0.26	0.36	0.35	−0.24	0.53	0.63	−0.42	0.45	**0.63**	−0.24	0.56	0.69	−0.40	0.47	**0.58**	−0.24	0.59	0.67	−0.54	0.50	**0.64**	−0.56
l_1-DL$_{\mathrm{CL}}$	0.45	0.58	−0.20	0.35	0.58	−0.20	0.53	0.64	−0.34	0.44	0.57	−0.20	0.56	0.69	−0.46	0.47	0.51	−0.20	0.57	0.67	−0.47	0.48	0.53	−0.43
**l_1-DL$_{\mathrm{PCA}}$**	0.47	**0.63**	−0.19	**0.37**	**0.60**	−0.21	**0.57**	**0.65**	−0.39	0.47	0.59	−0.21	0.56	**0.70**	−0.41	0.50	0.54	−0.21	**0.60**	**0.69**	−0.49	**0.51**	0.58	−0.46

Table S2: Average performance of each dFC state identification method using four different window lengths: 12 TRs (30.0 s), 18 TRs (45.0 s), 21 TRs (52.5 s) and 24 TRs (60.0 s). l_1-DL$_{\mathrm{PCA}}$ outperformed all the other methods in most cases, being only surpassed by other DL variants, and never by *k*-CL or PCA.

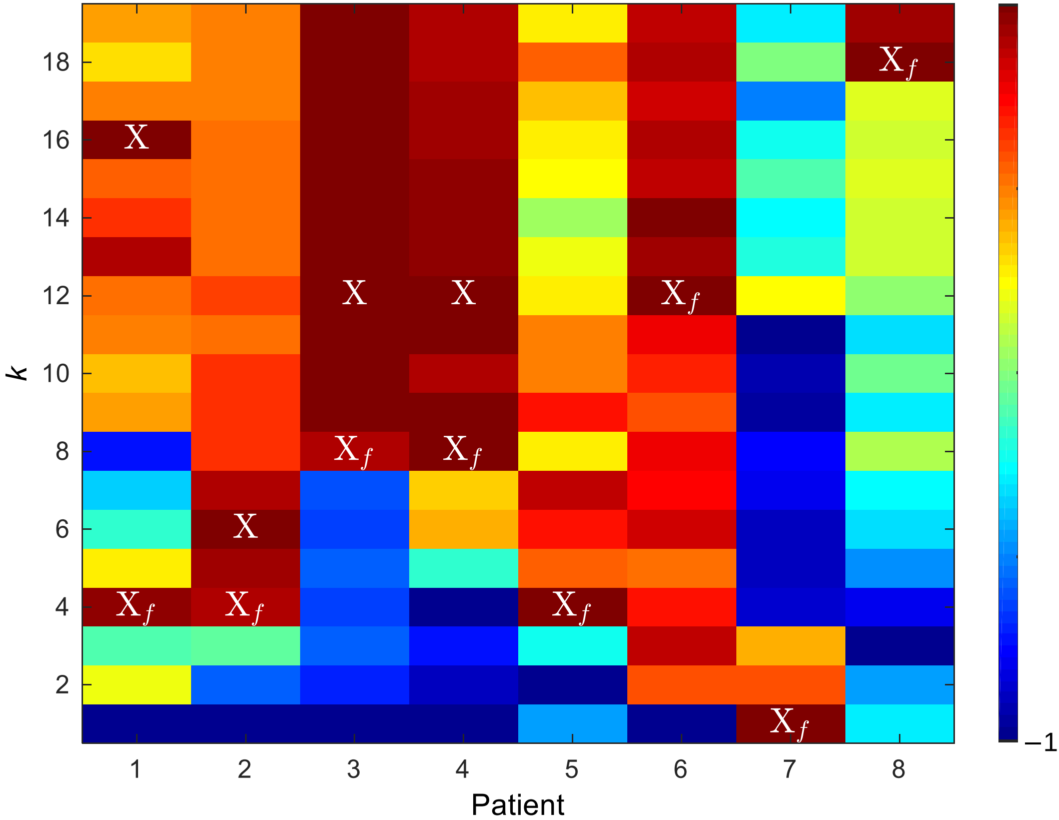

Figure S1: Correlation coefficients (ρ) obtained with l_1-DL$_{PCA}$ for each *k* and each patient. "X" highlights the *k* for which ρ_{max} was obtained, and "X_f" denotes the smallest *k* necessary to achieve 90% of ρ_{max}. These values were normalized between −1 and 1 patient-wise, for visualization purposes.
